# Implementation science made too simple: a teaching tool

**DOI:** 10.1186/s43058-020-00001-z

**Published:** 2020-02-25

**Authors:** Geoffrey M. Curran

**Affiliations:** 1 University of Arkansas for Medical Sciences, 4301 W. Markham St., #522-4, Little Rock, AR 72205 USA; 2 Central Arkansas Veterans Healthcare System, 4300 W 7th St, Little Rock, AR 72205 USA

**Keywords:** Implementation science, Implementation strategies, Implementation outcomes, Education

## Abstract

**Background:**

The field of implementation science is growing and becoming more complex. When teaching new learners, providing a clear definition of implementation science and a description of “its place” among related fields can be difficult. The author developed a teaching tool using very simple language to help learners grasp key concepts in implementation science.

**The teaching tool:**

The tool consists of a slide (visual aid) which provides simple and jargon-free definitions of implementation science, implementation strategies, and implementation outcomes, as well as a description of how implementation science relates to “effectiveness” research focusing on clinical/preventive interventions.

**Conclusion:**

The tool could be useful to new students in the field, as well as other scholars or stakeholders in need of a brief and plain language introduction to key concepts in implementation science.

Contributions to the literature

- The article provides a teaching tool to assist learners in implementation science to grasp key concepts in the field.
- The tool provides simple and jargon-free definitions of implementation science, implementation strategies, and implementation outcomes, as well as a description of how implementation science relates to “effectiveness” research focusing on clinical/preventive interventions.
- The tool is unique in its use of very simple language, and hence, it can use used with both scientists and non-scientists in need of a quick introduction to implementation science.

## Introduction

Implementation science can be complicated and at times even overwhelming. While the field is still considered “young,” implementation scientists have been hard at work developing frameworks, testing implementation strategies, and establishing implementation outcome measures. As a result, learners participating in introductory didactics on implementation science are often confronted with a dizzying array of information and recommendations to consider when thinking about or planning an implementation study. For example, Tabak et al. [1] identified 61 dissemination and/or implementation theories/frameworks/models available to help craft an implementation study. Just one of those frameworks, Damschroder et al.’s [2] widely used Consolidated Framework for Implementation Research (CFIR), offers 39 implementation factors to consider. Powell et al. [3] conceptualized 73 discrete implementation strategies available for consideration when developing an implementation intervention. Proctor et al. [4] offer 17 potential outcome domains to consider for an implementation study, and the Society for Implementation Research Collaboration has compiled a repository [5] of over 400 implementation-related measures.

**Fig. 1:**
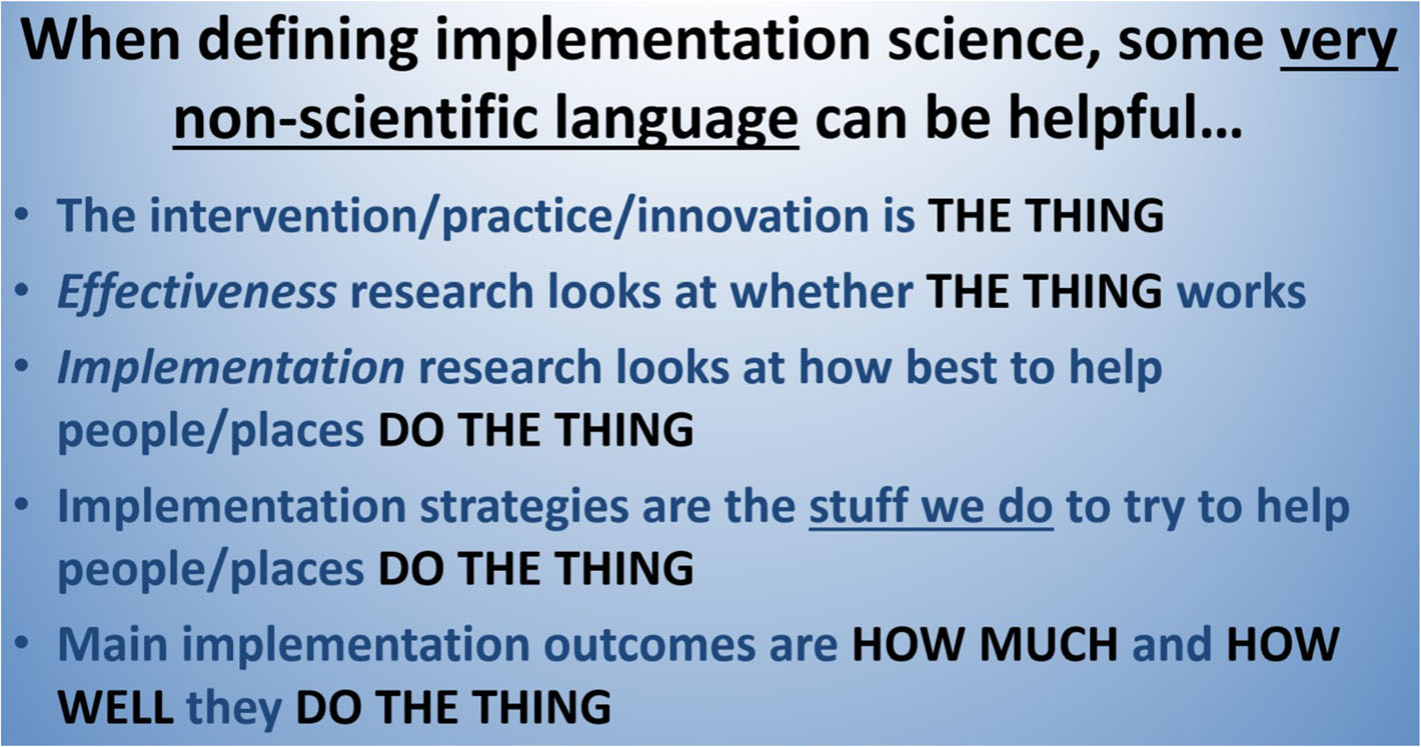
The slide used for the past 2 years

I have been teaching and lecturing in this field over 15 years, and I have presented the above information, and more, many times to learners in my own graduate implementation science course and in various seminars/workshops at other institutions. As a director of an academic center focused on implementation research, I have also provided dozens of consultations to researchers from other fields who are interested in exploring how best to implement interventions they have developed and tested. Recently, I have been searching for a way to quickly capture the essence of what implementation science is, what it is trying to do, and how it relates to the “clinical” or “effectiveness” research that often precedes it, *without intimidating jargon*. In my own course, I was getting the sense that I needed to start off, in the very first session, with a simpler definition and explanation than what I had been using. In consultations, I realized that I needed a simple way of defining and differentiating implementation science from what those scientists had already been doing. Over about a year’s time, I experimented with using very (*very*) simple language to get these points across. My goal was to keep it to one slide, and Fig. 1 shows the slide I have been using for the past 2 years.

## The teaching tool

Central to the logic inherent in the slide is the notion of an intervention/practice/innovation, referred to as “the thing,” whose implementation is in need of support. After I introduce “the thing,” I then explain that effectiveness research (which most of my students and consultees are already familiar with) is focused on whether “the thing” *works*—meaning that receiving it results (or not) in positive impacts on clinical/prevention/whatever outcomes. Next, I explain that implementation science/research focuses on *how best* to “do the thing.” This is my attempt at boiling down a more detailed definition [6] into the simplest and most basic of language. Next, I introduce the notion of implementation strategies, which I frame verbally as the *interventions that implementation scientists develop and test* to improve uptake of “the thing.” In keeping with the theme of using very simple language, I refer to implementation strategies on the slide as *the stuff we do* to try to help people and places to “do the thing.” Lastly, I introduce the notion that implementation science has its own primary outcome measures, distinct from the clinical/preventive outcomes used in effectiveness research. I refer to those outcomes as measures of *how much* and *how well* they (implementers) “do the thing.” Verbally, I explain that these measures are focused on the extent (how much) and the quality (how well) of implementation.

## Discussion

Since using the slide and the concepts of *the thing* and *do the thing* repeatedly in teaching, consultations, and everyday conversations, my students and local colleagues have adopted this terminology. After using the slide in numerous presentations at other institutions over the past 2 years (mostly centered on effectiveness-implementation hybrid designs, wherein this language can be especially helpful), many colleagues have used the slide and/or its concepts in their own teaching. For example, colleagues used these concepts during workshops/presentations at the 11th Annual Conference on the Science of Dissemination and Implementation in Health in Washington DC, December 2018 [7], and the Implementation Science Masterclass at King’s College, London, July 2019 [8]. This article is my attempt to share the slide and concepts as teaching tools more widely.

I wish to be clear that the slide, as is, has its limitations. It ignores the concept of de-implementation. It has an implicit focus/bias on *interventional* implementation science. And it certainly lacks detail. While that is also perhaps its greatest strength, it is worth noting that the slide does not “speak for itself.” I recommend it be presented by someone with expertise in implementation science who can provide context and linkage to the more “science-y” terms we normally use when describing and defining our science.

## Conclusion

Given the complexity of implementation science, providing a clear definition of it and a description of “its place” among related fields can be difficult. I developed this tool to assist my own teaching of students and other scholars new to implementation science. It has been useful in that regard. Further, the concepts of *the thing* and *do the thing* have also been helpful in providing a quick explanation of implementation science to non-scientists. So, feel free to try these ideas with others outside academia as well.

## Data Availability

Not applicable.
